# Co-activation of hedgehog and AKT pathways promote tumorigenesis in zebrafish

**DOI:** 10.1186/1476-4598-8-40

**Published:** 2009-06-25

**Authors:** Bensheng Ju, Jan Spitsbergen, Christopher J Eden, Michael R Taylor, Wenbiao Chen

**Affiliations:** 1 Vollum Institute, Oregon Health and Science University, Portland, Oregon 97239, USA; 2 Department of Molecular Physiology & Biophysics, Vanderbilt University Medical Center, Nashville, Tennessee, 37232, USA; 3 Center for Fish Disease Research, Oregon State University, Corvallis, Oregon 97331, USA; 4 Department of Developmental Neurobiology, St Jude Children's Research Hospital, Memphis, Tennessee 38105, USA; 5 Department of Chemical Biology & Therapeutics, St Jude Children's Research Hospital, Memphis, Tennessee 38105, USA

## Abstract

The zebrafish has become an important model for cancer research. Several cancer models have been established by transgenic expression of human or mouse oncogenes in zebrafish. Since it is amenable to efficient transgenesis, zebrafish have immense potential to be used for studying interaction of oncogenes and pathways at the organismal level. Using the *Gal4VP16-UAS *binary transgenic expression approach, we established stable transgenic lines expressing an EGFP fusion protein of an activated zebrafish Smoothened (Smoa1-EGFP). Expression of the zebrafish Smoa1-EGFP itself did not lead to tumor formation either in founder fish or subsequent generations, however, co-expressing a constitutively active human AKT1 resulted in several tumor types, including spindle cell sarcoma, rhabdomyoma, ocular melanoma, astrocytoma, and myoxma. All tumor types showed GFP expression and increased Patched 1 levels, suggesting involvement of zebrafish Smoa1 in tumorigenesis. Immunofluorescence studies showed that tumors also expressed elevated levels of phosphorylated AKT, indicating activation of the PI3K-AKT pathway. These results suggest that co-activation of the hedgehog and AKT pathways promote tumorigenesis, and that the binary transgenic approach is a useful tool for studying interaction of oncogenes and oncogenic pathways in zebrafish.

## Findings

**Figure 1:**
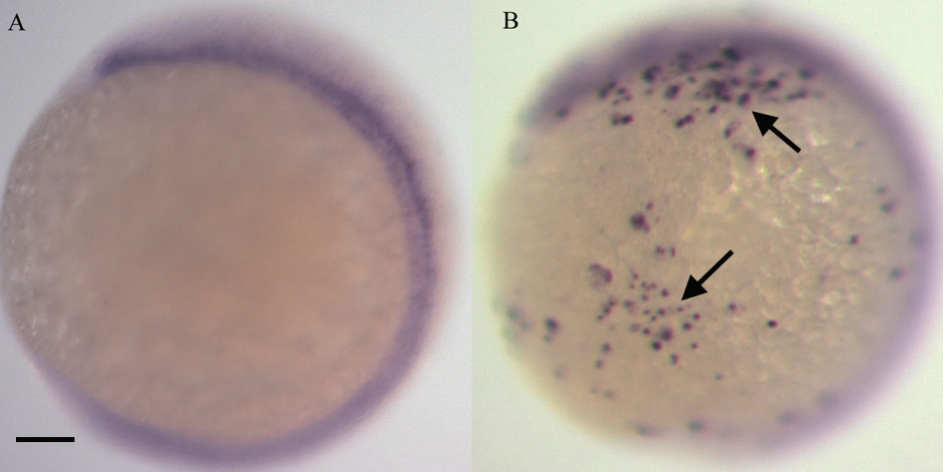
**Expression of zebrafish Smoa1 activated the Hh pathway**. A, whole-mount *in situ *hybridization of a control 12 hpf embryo showing *ptc1 *expression in adaxial structures. B, a 12 hpf transgenic embryo expressing *CMV-smoa1-EGFP *showed ectopic expression of *ptc1 *(Arrows). Scale bars, 100 μm.

The Hedgehog (Hh) pathway is involved in cell fate determination and embryonic patterning during early vertebrate development, and is also implicated in tumorigenesis [1]. Activation of the Hh pathway underlies the majority of sporadic human basal cell carcinoma (BCC) [2]. Expression of sonic Hedgehog, constitutively active Smoothened, and transcription factors Gli1 and Gli2 in keratinocytes results in BCC in transgenic frog, mice, and human skin [3-6]. Since zebrafish have emerged as a promising vertebrate system to model human cancers [7-9], we decided to determine whether activation of the Hh pathway in transgenic zebrafish could render them prone to developing BCC. We generated the zebrafish version of activated *Smoothened *using site-specific mutagenesis of wild type smoothened cDNA [Smo^W514L^, referred as Smoa1 hereafter, see Additional file 1]. To facilitate observation of the tumorigenesis processes, we tagged the zebrafish Smoa1 with C-terminal EGFP. We expressed it under the control of a *CMV *promoter, and observed GFP expression by 5 hours post fertilization (hpf). To assess the effect of Smoa1-EGFP expression on the Hh signalling pathway, we analyzed the expression of *patched 1 *(*ptc1*) [10], a marker for Hh activity, by *in situ *hybridization. We detected ectopic expression of *ptc1 *(n = 30) in the *Tg(CMV:smoa1-EGFP) *injected, but not in non-injected control embryos (Fig. 1A, 1B respectively), indicating Smoa1-EGFP could still activate the Hh pathway.

We further generated a binary transgenic construct based on the Gal4VP16-UAS system [11] [see Additional file 2]. The zebrafish *cytokeratin 4 (krt4) *promoter was used to drive expression of Gal4VP16 as it has been shown to direct EGFP expression exclusively in epithelial cells [12]. Injection of the *Tg(krt4:Gal4VP16;14 × UAS:smoa1-EGFP) *construct into 1-cell stage embryos led to observable levels of EGFP expression starting at about 5 hpf. We analyzed more than 100 founder fish over a period of one and a half years, but did not find any tumor. Adult founder fish were crossed to wild-type fish, and two male founder fish were found to carry the transgene in their germline. These lines were designated as line A and line B. Both lines did not show observable EGFP expression, but *in situ *hybridization using a *GFP *antisense probe detected low levels of GFP expression in skin epithelial cells [see Additional file 3]. Both lines were also crossed with a *Tg(14 × UAS:tdTomato) *reporter line that we generated in the *rose *background (unpublished results) to verify their expression patterns [13]. Line A showed epithelial restricted expression [see Additional file 3], while line B showed patchy epithelial expression and ectopic expression in neuronal cells of the brain, spinal cord, and trunk muscles (Data not shown). Fish positive for tdTomato expression in both lines were raised to adulthood and no tumor were found even when the fish reached one and a half years old. To further characterize the expression pattern for line A, an adult fish at two months old was sacrificed and paraffin sections were used for immunofluorescence against GFP. Expression was found predominantly in the skin epithelia [see Additional file 3], which was expected since the promoter driving the transgenic expression was a skin-specific *krt4 *promoter. Expression of GFP was also found in the retina ganglion cell layer [see Additional file 3]. We did not find prominent expression of GFP in other tissues or organs.

**Figure 2:**
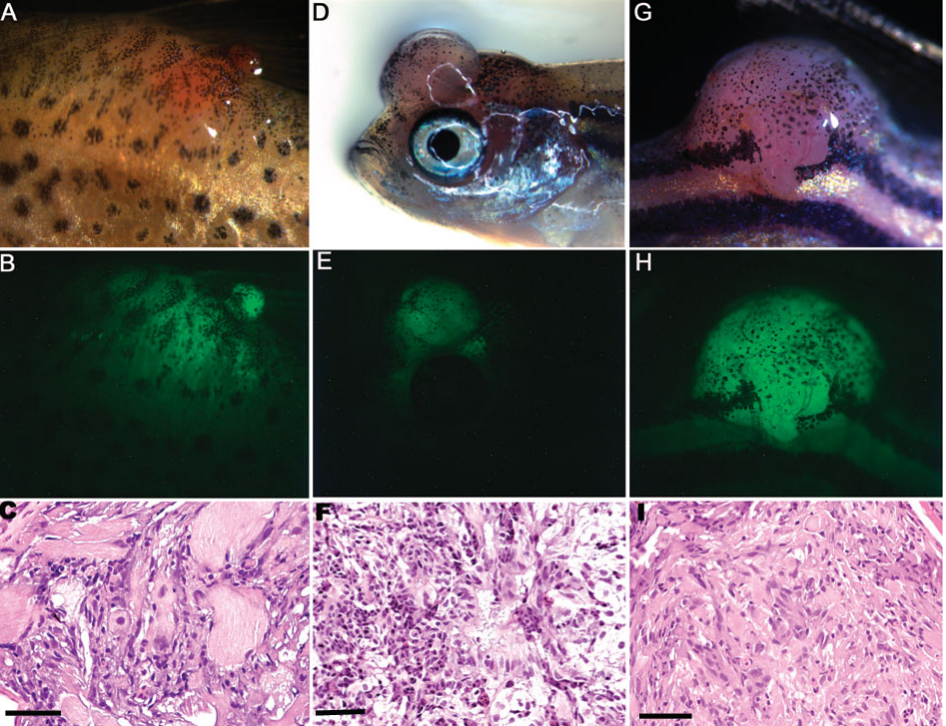
**Zebrafish tumors induced by co-expression of zebrafish Smoa1 and constitutively active human AKT1**. A, B and C, a 12-week-old fish with rhabdomyoma (A), showing GFP expression exclusively in the tumor (B); D, E and F, a 6-week-old fish with glioblastoma; G, H and I, a 8-week-old double transgenic fish with astrocytoma in the lower trunk region. Scale bars, 50 μm.

Since the *Tg(krt4:Gal4V16;14 × UAS-smoa1:EGFP) *fish did not develop tumors at one and a half years of age, we decided to test whether other oncogenes could collaborate to promote tumorigenesis. We chose to use transgenic line A as it showed rather specific expression in epithelia in comparison to line B. A recent report has shown that PI3K and AKT are essential for SHH signalling [14]. As a first test of our co-expression strategy, we expressed the wild-type and the constitutively active human *AKT1 *by injecting *Tg*(*UAS:hAKT1) *or *Tg(UAS:myrhAKT1) *plasmid DNA into progeny of F1 siblings of the *Tg(krt4:Gal4V16;14 × UAS-smoa1-EGFP) *line A fish. Both the *smoa1 *and *AKT1 *genes were under control of the *14 × UAS *promoter, so their expression was driven by the same *krt4 *promoter through Gal4VP16. While no tumor was found in the resultant F2 fish injected with *Tg*(*UAS:hAKT1*), various tumor types in the trunk, the eye, and the head region were observed in the F2 fish injected with *Tg(UAS:myrhAKT1)*. The tumor types identified include a case of spindle cell sarcoma, rhabdomyoma, ocular melanoma, myoxma, and several cases of astrocytoma and glioblastoma (Fig. 2 and Table 1). These tumor types were diagnosed according to histological criteria used in mammals [15]. GFP expression was found exclusively in the tumors, due to either the additive effect of a large number of weakly EGFP-positive cells or the loss of mechanisms that suppress Smoa1-EGFP levels, or both. In any case, the detection of EGFP expression in tumors indicated the involvement of the zebrafish Smoa1 in tumorigenesis (Fig. 2B, 2E). Overall, nearly 10% of injected fish developed tumors before reaching 3 months of age. Surprisingly, the rest of the fish were tumor-free at 18 months of age, at which we stopped the experiment. We also identified fish that carried both zebrafish *smoa1 *and the oncogenic human *AKT1 *in their germline. We crossed two of these fish to each other, and found 3 out of 15 of the offspring developed tumors at around 2 months of age, including one case of astrocytoma in the lower-trunk region (Fig. 2H), the other two cases were not diagnosed. In contrast, expression of either wild-type or constitutively active human AKT1 alone, driven by the same *krt4 *promoter, did not induce tumors. In fact, even when we expressed the constitutively active human AKT1 driven by a strong ubiquitous *β-actin *promoter, no tumor was found in more than 50 fish over an 18 months period. Thus, the tumors likely resulted from collaboration between the constitutively active hAKT1 and Smoa1.

**Table 1:** Tumors induced by co-expression of zebrafish *smoa1 *and constitutively active human *AKT1*

Tumor location (cases)	Fish age (weeks)	GFP expression	Elevated phospho-AKT1	Diagnoses
Trunk (4)	3	Yes	ND	Rhabdomyoma
	4	Yes	Yes	ND
	6	Yes	ND	ND
	12	Yes	ND	Rhabdomyoma

Eye (3)	4	Yes	ND	Ocular Melanoma
	12	Yes	Yes	Astrocytoma
	12	Yes	ND	Astrocytoma

Head (4)	6	Yes	ND	Glioblastoma
	6	Yes	ND	Glioblastoma
	8	Yes	ND	ND
	12	Yes	ND	Astrocytoma

Others (2)	4	Yes	ND	Myxoma
	8	Yes	ND	Spindle cell sarcoma

*The tumors were found from 4 batches of injection involving 147 injected F2 fish that had survived beyond 2-week-old. Fish were observed for one and a half years, no other tumors were found in fish more than 3 months of age. ND: Not determined.

**Figure 3:**
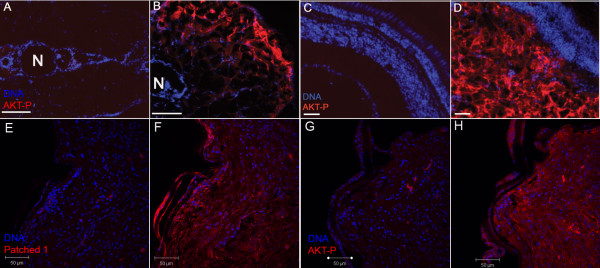
**Elevated phospho-AKT1 and Patched 1 levels in zebrafish tumors**. A, B, a 3-week-old transgenic fish with trunk tumor (B) and its age-matched tumor-free fish (A). C, D, a 12-week-old fish with an eye tumor (D) and its age-matched tumor free fish (C). Immunofluorescence was done on cryosections for the above fish. E-H, an astrocytoma from a double transgenic fish showed elevated levels of both Patched 1 (F) and phosphorylated AKT (H). Immunofluoresence was done on paraffin sections for this tumor. Negative controls for Patched 1 (E) and phosphorylated AKT (G) were treated the same way except no primary antibodies were added. N, Notochord; AKT-P, phosphorylated AKT. Scale bars, 100 μm for A-D, 50 μm for E-H.

Activation of the PI3K-AKT pathway leads to phosphorylation of AKT1 [14]. To determine whether phospho-AKT1 levels were increased in the tumors, we performed immunofluorescence studies on a 3-week-old fish with a trunk tumor showing strong GFP expression and on a 12-week-old fish with an ocular tumor showing weak GFP expression. Immunofluorescence analysis indicated that both tumors had significantly higher levels of phosphorylated AKT (Fig. 3B, 3D) when compared to age-matched, tumor-free transgenic fish (Fig. 3A, 3C). To demonstrate that both the SHH and AKT pathways were activated in a same tumor, paraffin sections of the astrocytoma as shown in Fig. 2G was used for immunofluorescence study against Patched 1 and phosphorylated AKT. As shown in Fig. 3F and 3G, the tumor had elevated levels of both Patched 1 and phosphorylated AKT, indicating that both pathways were activated in this tumor. These results suggest that the tumors resulted from collaborative expression of both the zebrafish Smoa1 and the constitutively active human AKT1.

Here we demonstrated that several tumor types in zebrafish can be induced by transgenic expression of oncogenes, most likely due to co-expression of an oncogenic zebrafish Smoa1 and the constitutively active human AKT1. It was surprising that we did not find any skin tumor, as activation of either the Hh or AKT pathway [16,17] leads to skin hyperplasia and skin tumors in mouse. We also generated a transgenic line using the Tet-Off system that expressed the Smoa1-EGFP driven by a 5 kb zebrafish *cytokeratin 5 *(*krt5*) gene promoter. This transgenic line expressed higher levels of zebrafish Smoa1, but we did not find any skin tumor in fish that reached two years of age (Data not shown). It is possible that either: 1) the level of Smoa1 expression was below the threshold for tumorigenesis; 2) the Smoa1-EGFP fusion protein was not potent enough; 3) the *krt4 *and *krt5 *promoters were not active in keratinocyte progenitors; or 4) the combinations thereof. Alternatively, zebrafish skin may be quite refractory to developing basal cell carcinoma in comparison to frog and mammals, due to differences either in structure or in tumorigenic mechanisms. As for the other tumor types, especially the neuronal tumors, aberrant activation of the Hh pathway has been implicated in medulloblastoma and glioma [18]. Studies on AKT pathway implicate it not only in skin tumors, but also in glioblastoma [19]. Furthermore, the Akt signalling pathway contributes to SHH-induced medulloblastoma formation [20]. Therefore, it is conceivable that tumor types of neuronal origin may be induced in fish. But how exactly these tumors were induced in our transgenic fish is unknown. Immunofluorescence study showed that transgenic fish expressed the fusion protein not only in skin epithelia, but also in retina, which was not found in a previous study using the same promoter. An enhancer-trapping event might have occurred that led to ectopic expression of low levels of zebrafish Smoa1 in neuronal progenitor cells, which made the cells prone to developing tumors. Expression of a second oncogene, the constitutively active human AKT1, in the same cell types eventually led to tumorigenesis. It is also possible that integration of the transgenic construct into the zebrafish genome somehow activated an endogenous oncogene or inactivated a certain tumor suppressor gene, therefore contributed to tumor formation. We tried to determine the insertion copy numbers and the insertion sites in Line A fish through linker-mediated PCR and inverse PCR. We only detected one insertion, which was integrated at an intergenic region on zebrafish chromosome 11 (Data not shown). We do not know the effects of this integration on tumorigenesis in this particular transgenic line.

Cancers result from progressive accumulation of mutations in multiple cancer genes [21,22]. How different cancer genes interact with each other leading to different types of cancer is still a challenging subject. So far, oncogenic interactions have largely been studied in cultured cells or in mouse models. As a complement to these studies, the zebrafish provides a powerful model system to study interactions between different cancer genes and pathways at the organismal level because it is amenable to highly-efficient and cost-effective transgenic strategies [23,24]. Binary transgenic technology, such as the Gal4VP16-UAS system, the Tet-On and Tet-Off system [25], and the mifepristone inducible LexPR system [26] work efficiently in zebrafish, making it possible to express multiple oncogenes in the same tissue or cell type under the control of the same non-endogenous transcription factor. A limitation for using these binary transgenic approaches, as we have learned from our experience, is that stable lines carrying oncogenes may have diminished survivability. Therefore, their potential to be used for collaborative studies with other oncogenes may be somewhat compromised. Another challenge will be to untangle the role of individual oncogenes in the tumor formation and progression processes.

In conclusion, we provide *in vivo *evidence that co-expression of the zebrafish Smoa1 and the constitutively active human AKT1 lead to tumorigenesis in zebrafish, establishing that the binary transgenic approach is a useful tool for studying collaboration between oncogenes and oncogenic pathways in the zebrafish model.

## Materials and methods

### Zebrafish and maintenance

Zebrafish (*Danio rerio*) were maintained in Aquatic Habitats systems (Apopka, FL) on a 14 to 10 light dark cycle using a protocol approved by OHSU IACUC. The *leopard long fin *(TL), and the *rose *mutant fish which has a mutation in the *endothelial receptor b1 *gene causing light pigmentation were used. Embryos were reared in 0.3× Danieau's solution at 28.5°C

### DNA constructs and microinjection

The transgenic DNA constructs are based on either the miniTol2 vector (denoted T2) or the I-Sce I meganuclease vector (denoted I) [see additional file 1]. The *Tg*(*krt4:Gal4VP16;14 × UAS:smoa1-EGFP*) transgenic construct contains a 2.2 kb zebrafish *krt4 *promoter, the Gal4VP16-UAS sequence from pEF-GVP-UG, and coding sequence for an activated zebrafish smo fused to EGFP at the C-terminus. The I*U-mCherry *plasmid, which contains 14 × UAS and E1b sequence from pUG and mCherry coding sequence, is the basic vector from which other UAS-driven vectors are derived, including pIU-hAKT1 and pIU-myrhAKT1 (myristylated human AKT1 lacking the Pleckstrin Homology Domain sequence, referred to as constitutively active human AKT1). To deliver transgenes for transient expression or to establish stable lines using Tol2-based plasmids, about 1 nl sterile isotonic saline solution containing transgene DNA (30 ng/μl) and transposase RNA (30 ng/μl) was injected into fertilized eggs of the leopard strain. For the meganuclease-based constructs, the vector was mixed with *I-SceI *meganuclease and 30 pg of plasmid in 1 nl of the mixture was injected. Transgenic lines were identified by PCR using a *zebrafish smoothened *forward primer (5'-GGAAAGGAACAAACTTTGGATG-3') and an *EGFP *reverse primer (5'-CTGAACTTGTGGCCGTTTACGTC-3').

### Histological study of zebrafish tumors

Zebrafish with tumors were fixed in 4% paraformaldehyde at 4°C for 24–48 hours, washed with PBST and decalcified in 0.5 M EDTA for up to 7 days depending on size. Tissues were dehydrated in a series of graded ethanol solutions and xylene, then embedded in paraffin. Orientation of the fish depended on location of the lesions. For fish with no gross lesions, individuals were cut in half sagittally just to the left of midline and both halves of the fish were placed into the cassette for sectioning. For small fish, serial sections were prepared. Sections were 4–6 μm thick and were stained with hematoxylin and eosin. Since standardized diagnostic criteria for fish tumors is not yet available, diagnostic criteria for other species, such as human and mouse were used to identify zebrafish tumors.

### Immunofluorescence study of zebrafish tumors

For cryosections, zebrafish with tumors were fixed in 4% paraformaldehyde at 4°C for 24 hours, washed with PBST. Tumor tissues were first immersed in 15% glucose for 1 hour, followed by immersion in 30% sucrose overnight. They were then embedded in O.C.T compound and sectioned at 12 μm thickness. For paraffin sections, fish were treated as mentioned in histological study of zebrafish tumors. A rabbit anti-GFP polyclonal antibody (Invitrogen, 1:500), a rabbit anti-phospho-AKT (Thr308) antibody (C31E5, Cell Signalling Technology, 1:200), and a goat anti-Patched 1 (zebrafish) antibody (Everest Biotech, 1:200) were used for immunofluorescence studies of the tumors. Images were acquired with an inverted Zeiss microscope equipped with a CCD camera and AxioVision software. All images are processed in Photoshop.

## Abbreviations

PI3K: Phosphoinositide 3-kinases; AKT1: v-akt murine thymoma viral oncogene homolog 1; UAS: upstream activation sequence; CMV: Cytomegalovirus.

## Competing interests

The authors declare that they have no competing interests.

## Authors' contributions

BJ and WC conceived, planned the experiments. BJ made the transgenic constructs and generated transgenic fish. JS identified the tumors. CJE and MRT did immunofluorescence studies on tumors. All authors read and approved the manuscripts.

## Supplementary Material

Additional file 1 **Amino acid sequence alignment of mouse and zebrafish Smoothened**. The alignment indicated that the W514 of zebrafish Smoothened is the W539 equivalent of mouse Smoothened. It was mutated to L in the active forms (highlighted in red). Accession numbers for zebrafish and mouse Smoothened are [NP_571102] and [NP_795970], respectively. Click here for file

Additional file 2 **Overall strategy for co-expression of oncogenes in zebrafish**. Stable transgenic lines expressing zebrafish Smoa1 were generated using a Tol2-based vector (A). Constitutively active human AKT1 (myrhAKT1) was incorporated into a meganuclease-based vector (B). The zebrafish *krt4 *promoter could simultaneously activate *smoa1-EGFP *and *myrhAKT1 *expression through Gal4VP16-UAS. Click here for file

Additional file 3 **Expression patterns of transgenic line A**. The data indicated that the *cytokeratin 4 *promoter drove epithelial cells-specific expression (arrows) of smoa1-EGFP as shown by *in situ *hybridization against EGFP in 12 hpf F1 embryos (A), and of tdTomato in a 24 hpf embryo generated by crossing the *Tg*(*krt4:Gal4VP16;14 × UAS:smoa1-EGFP*) and *Tg(UAS:tdTomato) *transgenic fish (B). At adult stage, GFP was detected predominantly in skin epithelial cells (C, arrow) and the retinal ganglion cells (D, arrow). Click here for file
