# Supplementary material for: Co-activation of hedgehog and AKT pathways promote tumorigenesis in zebrafish
Source: Mol Cancer. 2009 Jun 25;8:40. doi: 10.1186/1476-4598-8-40 (PMC2711045; doi:10.1186/1476-4598-8-40)
Supplement: Additional file 1 — Amino acid sequence alignment of mouse and zebrafish Smoothened. The alignment indicated that the W514 of zebrafish Smoothened is the W539 equivalent of mouse Smoothened. It was mutated to L in the active forms (highlighted in red). Accession numbers for zebrafish and mouse Smoothened are [NP_571102] and [NP_795970], respectively. [file 1476-4598-8-40-S1.doc]

**Bensheng Ju Supplementary Fig. S1**

**Zebrafish** --------------------------MSSKRPCSIVGSFWMLWIWTATSMVARAVILHPN

**Mouse** MAAGRPVRGPELAPRRLLQLLLLVLLGGPGRGAALSGNVTGPGPHSASGSSRRDVPVTSP

**Zebrafish** ETIFNDFCKKSTTCEVLKYNTCLGSPLPYTHTSLILAEDSETQEEAFEKLAMWSGLRNAP

**Mouse** PPPLLSHCGRAAHCEPLRYNVCLGSALPYGATTTLLAGDSDSQEEAHGKLVLWSGLRNAP

**Zebrafish** RCWAVIQPLLCAVYMPKCENGKVELPSQHLCQATRNPCSIVERERGWPNFLKCENKEQFP

**Mouse**  RCWAVIQPLLCAVYMPKCENDRVELPSRTLCQATRGPCAIVERERGWPDFLRCTP-DHFP

**Zebrafish** KGCQNEVQKLKFNTSGQCEAPLVKTDIQASWYKDVEGCGIQCDNPLFTEDEHSDMHSYIA

**Mouse** EGCPNEVQNIKFNSSGQCEAPLVRTDNPKSWYEDVEGCGIQCQNPLFTEAEHQDMHSYIA

**Zebrafish** VFGTITLLCTFLHLATFLADWKNSNRYPAVILFYVNACFFIGSIGWLAQFMDGARNEIVC

**Mouse** AFGAVTGLCTLFTLATFVADWRNSNRYPAVILFYVNACFFVGSIGWLAQFMDGARREIVC

**Zebrafish** KSDNTMRLGEPSSTETLSCVIIFVIVYYSLMSGVIWFVMLTYAWHTSFKALGTTHQPLSG

**Mouse**  RADGTMRFGEPTSSETLSCVIIFVIVYYALMAGVVWFVVLTYAWHTSFKALGTTYQPLSG

**Zebrafish** KTSYFHLVTWSIPFILTVAILANSQVDADSVSGICFVGYRYYEYRAGFVLAPIGFVLVIG

**Mouse** KTSYFHLLTWSLPFVLTVAILAVAQVDGDSVSGICFVGYKNYRYRAGFVLAPIGLVLIVG

**Zebrafish** GYFLIRGVMTLFSIKSNHPGLLSEKAASKINETMLRLGIFGFLAFGFVLITFGCHFYDFF

**Mouse** GYFLIRGVMTLFSIKSNHPGLLSEKAASKINETMLRLGIFGFLAFGFVLITFSCHFYDFF

**Zebrafish**  NQAEWERSFREYVLCEANVTIAHQTNKPIPECAIKNRPSLLVGKINLFSMFGTGIAMSTW

**Mouse** NQAEWERSFRDYVLCQANVTIGLPTKKPIPDCEIKNRPSLLVEKINLFAMFGTGIAMSTW

**Zebrafish** VWTKATILIWKRTWFRIIGRSDDEPKRIKKSKMIAKAFSKRKELQKDPEKELSFSMHTVS

**Mouse** VWTKATLLIWRRTWCRLTGHSDDEPKRIKKSKMIAKAFSKRRELLQNPGQELSFSMHTVS

**Zebrafish** HEGPVAGINFDLNEPSIEMSSAWAQHVTKMVARRGAILPQDISVTPTGTPIPPPEERNKL

**Mouse** HDGPVAGLAFDLNEPSADVSSAWAQHVTKMVARRGAILPQDVSVTPVATPVPPEEQAN-M

**Zebrafish** WMVEAEISPEMMKRKKKKKKRRKEVRPAGPAADEGNPAYHRREFGPSAVPRLPKLPGHRS

**Mouse** WLVEAEISPELEKRLGRKKKRRKRKKEVCPLRPAPELHHSAPVPATSAVPRLPQLPRQKC

**Zebrafish** LVANLWEQQRQQQEEQDMLPGAFPEFRPSCPLPYQDRYGGLGYLRNKPSSLPLANPLTLR

**Mouse** LVAANAWGTGESCRQGAWTLVSNPFCPEPSPHQDPFLPG---------------------

**Zebrafish**  DSMQGDLSHFQQSSWQPKGVFRHLGQEASMMDVGRTAVVPRADGRRGVQIHSRTNLMDAE

**Mouse**  --------------------------------ASAPRVWAQGRLQGLGSIHSRTNLMEAE

**Zebrafish**  LLDADSDF

**Mouse** ILDADSDF
