# Supplementary figures and images for: Co-activation of hedgehog and AKT pathways promote tumorigenesis in zebrafish
Source: Mol Cancer. 2009 Jun 25;8:40. doi: 10.1186/1476-4598-8-40 (PMC2711045; doi:10.1186/1476-4598-8-40)

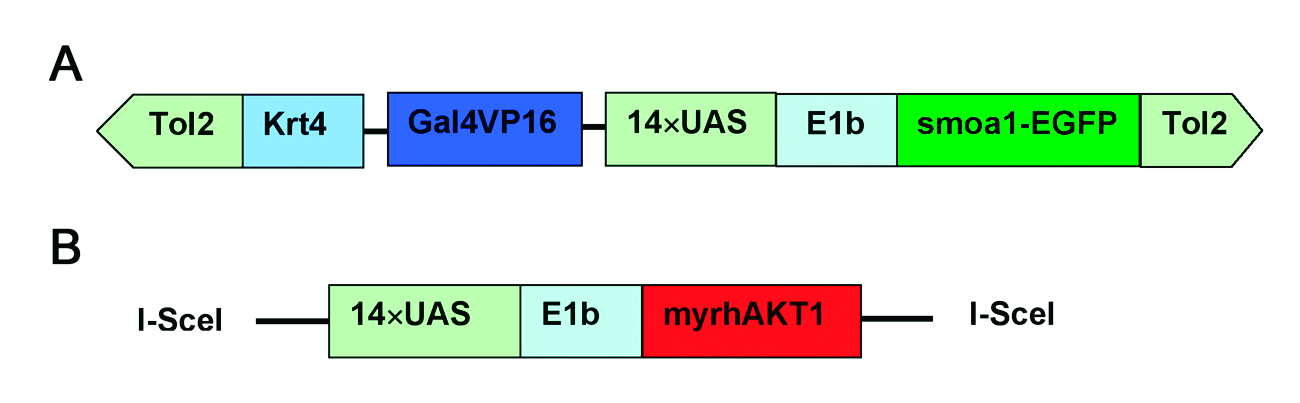

Supplement: Additional file 2 — Overall strategy for co-expression of oncogenes in zebrafish. Stable transgenic lines expressing zebrafish Smoa1 were generated using a Tol2-based vector (A). Constitutively active human AKT1 (myrhAKT1) was incorporated into a meganuclease-based vector (B). The zebrafish krt4 promoter could simultaneously activate smoa1-EGFP and myrhAKT1 expression through Gal4VP16-UAS. [file 1476-4598-8-40-S2.tiff]

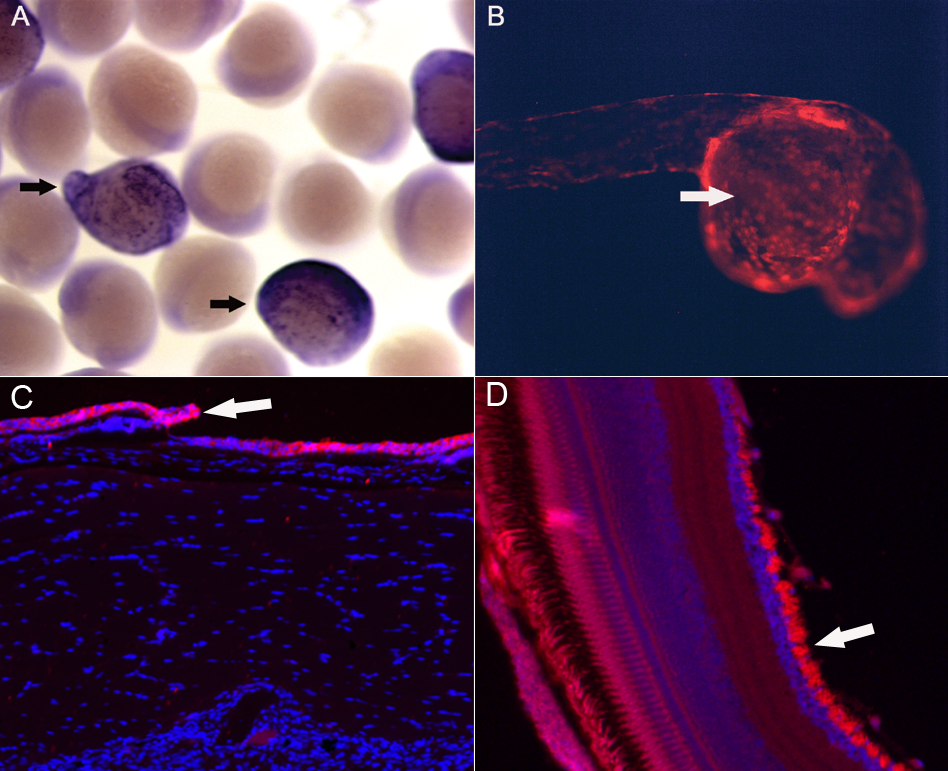

Supplement: Additional file 3 — Expression patterns of transgenic line A. The data indicated that the cytokeratin 4 promoter drove epithelial cells-specific expression (arrows) of smoa1-EGFP as shown by in situ hybridization against EGFP in 12 hpf F1 embryos (A), and of tdTomato in a 24 hpf embryo generated by crossing the Tg(krt4:Gal4VP16;14 × UAS:smoa1-EGFP) and Tg(UAS:tdTomato) transgenic fish (B). At adult stage, GFP was detected predominantly in skin epithelial cells (C, arrow) and the retinal ganglion cells (D, arrow). [file 1476-4598-8-40-S3.tiff]
